# Atomic-Scale Mapping of Interfacial Water on Oxide Surfaces via Proton-Resolved NMR and *Ab Initio* Simulations

**DOI:** 10.1021/jacs.5c18863

**Published:** 2026-03-16

**Authors:** Lorenzo Agosta, Ken Conover, Przemyslaw Rzepka, Alisa Gordeeva, Adam Slabon, Istvan Pelczer, Annabella Selloni, Kersti Hermansson, Aleksander Jaworski

**Affiliations:** † Department of Chemistry-Ångström, 8097 Uppsala University, Uppsala 751 21, Sweden; ‡ Department of Chemistry, 6740 Princeton University, Princeton, New Jersey 08544, United States; § 27219 J. Heyrovsky Institute of Physical Chemistry, Czech Academy of Sciences, Prague 18223, Czech Republic; ∥ Department of Chemistry, 111125 Stockholm University, Stockholm 106 91, Sweden; ⊥ Chair of Inorganic Chemistry, 26603 Bergische Universität Wuppertal, Wuppertal 42119, Germany

## Abstract

Understanding the molecular structure of water at solid–liquid interfaces is essential for advancing catalysis, energy conversion, and environmental technologies. However, directly characterizing interfacial water species in excess liquid water remains a major experimental challenge. Here, we introduce a new strategy that combines high-resolution ^1^H magic-angle spinning (MAS) nuclear magnetic resonance (NMR) spectroscopy with first-principles molecular dynamics simulations to resolve and assign the chemical environments of interfacial water and hydroxyl species on hydrated titanium dioxide (TiO_2_) nanoparticles. Using partial proton–deuteron exchange and fast MAS techniques, we achieve site-specific detection of surface-bound H_2_O and OH groups at the solid–liquid interface. This enables a detailed atomistic assessment of surface hydration states under ambient conditions. Our results reveal that the fully hydrated anatase (101) TiO_2_ surfaces are positively protonated and exhibit hydrophobic behavior, a counterintuitive finding with significant implications for interfacial reactivity. The approach developed in this work is widely applicable for unraveling complex hydration structures at oxide–water interfaces with molecular resolution.

## Graphical abstract

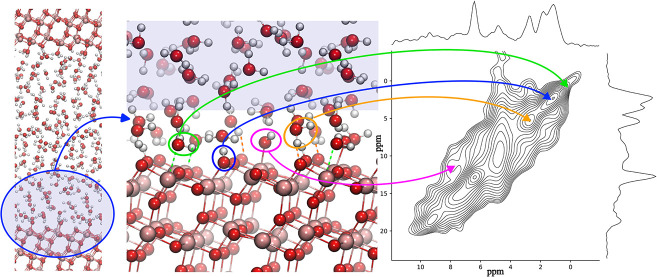

## Introduction

A wide range of surface-mediated processes, from heterogeneous catalysis and electrochemistry to environmental remediation and biomaterial design,^1,2^ involve solid surfaces in the presence of a significant excess of liquid water phase. Yet, direct detection and characterization of interfacial water species, particularly under full hydration and ambient conditions, remain a major experimental challenge. The low concentration of surface species and their dynamic nature are overwhelmed by the background of bulk water, which hinders molecular-level analysis. Thus, approaches capable of resolving these surface water structures with chemical specificity are urgently needed for progress in both fundamental and applied surface science.

Titanium dioxide (TiO_2_) is a prototypical metal oxide widely used to study aqueous–solid interfaces due to its importance in photocatalysis,^3^ antifogging coatings,^4^ and biointerfaces.^5^ The behavior of interfacial water on TiO_2_ is believed to play a central role in its photocatalytic activity.^91^ A variety of experimental techniques, such as inelastic and quasi-elastic neutron scattering (INS, QENS),^6–8^ scanning tunneling microscopy (STM),^9^ infrared (IR) spectroscopy,^10^ and sum-frequency generation (SFG),^11,12^ have provided indirect evidence of constrained or oriented water molecules near the surface. However, these approaches lack the resolution to resolve specific adsorbed species, such as hydroxyl groups and molecular water. In particular, it remains unclear how water behaves at fully hydrated surfaces: how it binds, whether it dissociates spontaneously, and whether surface hydration results in hydrophilic or hydrophobic behavior.

Atomistic simulations using molecular dynamics based on density functional theory (DFT-MD) have provided important insights into the structure and reactivity of water on TiO_2_ and other metal oxide surfaces and nanoparticles.^13,14^ For example, DFT-MD simulations predicted layered water near several TiO_2_ facets and increasing water mobility with distance from solid interfaces.^15^ Spontaneous water dissociation has been observed experimentally and by simulations in the low-coverage regime.^16–18^ Recent advances in machine learning algorithms have allowed DFT-MD simulations to be extended to time scales where water dissociation could be sampled on fully hydrated TiO_2_ surfaces.^19,20^ However, there has been no direct experimental validation of these interfacial structures in the presence of excess liquid water.

Solid-state nuclear magnetic resonance (NMR) spectroscopy, particularly fast ^1^H MAS NMR, is uniquely suited for probing disordered systems and low-concentration surface species due to its sensitivity to local chemical environments.^21^ Moreover, calculated NMR chemical shifts from quantum chemical simulations offer a powerful link between theory and experiment.^92^ Despite this potential, previous ^1^H MAS NMR studies of hydrated TiO_2_ ^22–24^ relied on conditions with limited (or non-excess) liquid water and/or yielded insufficient resolution in fully hydrated conditions to unambiguously resolve multiple distinct interfacial proton environments in the presence of a dominant liquid-water signal. This was probably due to the broadening of the signal caused by strong dipolar couplings between immobilized protons. Alternative approaches using dry samples have shown limited structural resolution.^25–29^ Recent ^1^H–^17^O 2D MAS NMR studies in enriched samples have allowed the observation of adsorbed water layers,^30^ but the ambiguity in proton chemical environments remains unresolved.

Here, we address this challenge by introducing a high-resolution ^1^H MAS NMR method enabled by selective proton–deuteron exchange to reduce dipolar broadening. This approach, adapted from biomolecular NMR protocols,^31–35^ dramatically improves spectral resolution and, as we will see, it allows us to resolve chemically distinct water and hydroxyl species at the fully hydrated TiO_2_–water interface. By combining this experimental approach with *ab initio* calculated chemical shifts from DFT-MD simulations of nano-surface–water systems, we achieve a direct chemically specific identification of interfacial water structures under realistic aqueous conditions. Our findings reveal a positively protonated TiO_2_ surface with intact isolated water molecules showing a hydrophobic character—a result that challenges the prevailing picture and offers a new platform for probing water–solid interfaces in material systems.

## Results

### Experiments

The most abundant polymorphs of TiO_2_ are rutile and anatase. The latter is more stable for nanoparticles smaller than 10 nm.^36^ In our NMR experiment, commercial TiO_2_ nanopowder (Sigma-Aldrich 718467) was used, and transmission electron microscopy (TEM) revealed a uniform nanoparticle size of ∼20 nm; see Figure 1a. The interplanar spacing as obtained from high-resolution transmission electron microscopy (HRTEM) images was ∼0.352 nm, which matches with that of anatase, with the majority (101) facet exposed. The reflection peaks obtained from the selected area electron diffraction (SAED) patterns (Figure 1b) can be indexed to the anatase phase of TiO_2_, which is in line with the Rietveld analysis of powder X-ray diffraction (XRD) data. The latter confirms that the anatase/rutile ratio is 88/12 (±1) (see Figure S1). Given the rather small amount of rutile detected by the X-ray analysis and the fact that its presence was not discernible at the particles’ surface in high-resolution TEM, we will not discuss this polymorph further.

**Figure 1:**
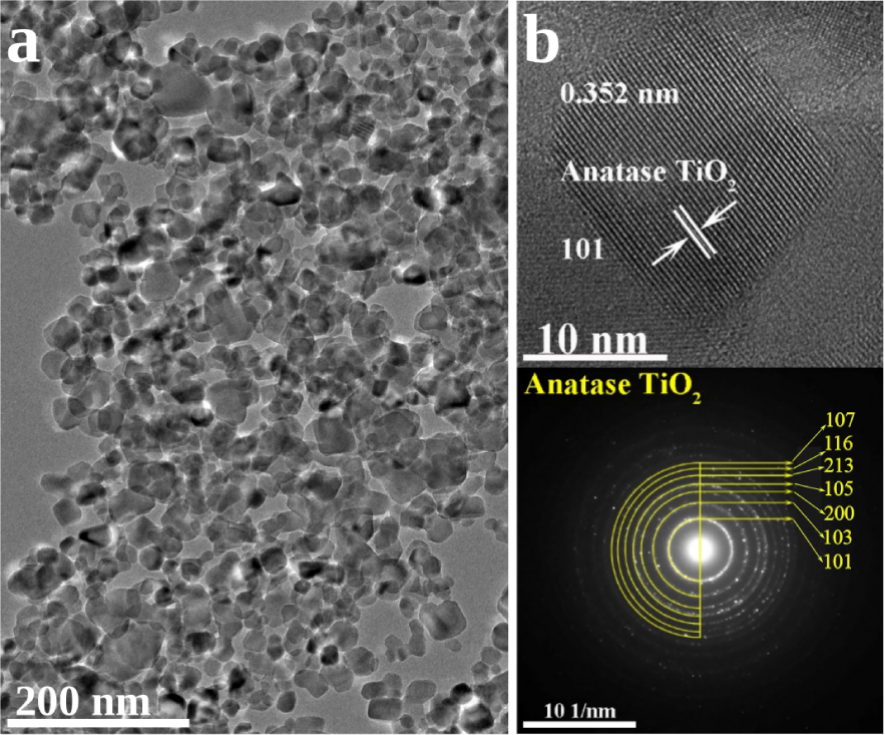
(a) TEM image of TiO_2_ nanoparticles used in our NMR experiments. (b) HRTEM image and SAED pattern revealing the anatase phase.

An MD snapshot of the anatase (101) surface in contact with liquid water is shown in Figure 2. On this surface, water molecules adsorb by forming dative bonds with 5-coordinated surface titanium atoms (Ti_[5]_) and strong hydrogen bonds with bridging oxygen atoms (O_br_). Adsorbed water at Ti_[5]_ can dissociate by transferring a proton to a neighboring O_br_, resulting in a terminal hydroxyl at Ti_[5]_ (Ti_[5]_–OH) and a bridging hydroxyl at an O_br_ (O_br_-H).^14^ The water molecules adsorbed on Ti_[5]_ sites are commonly assigned to the first hydration layer, while the water molecules hydrogen-bonded to the O_br_ sites constitute the second hydration layer.^12,15,37^

**Figure 2:**
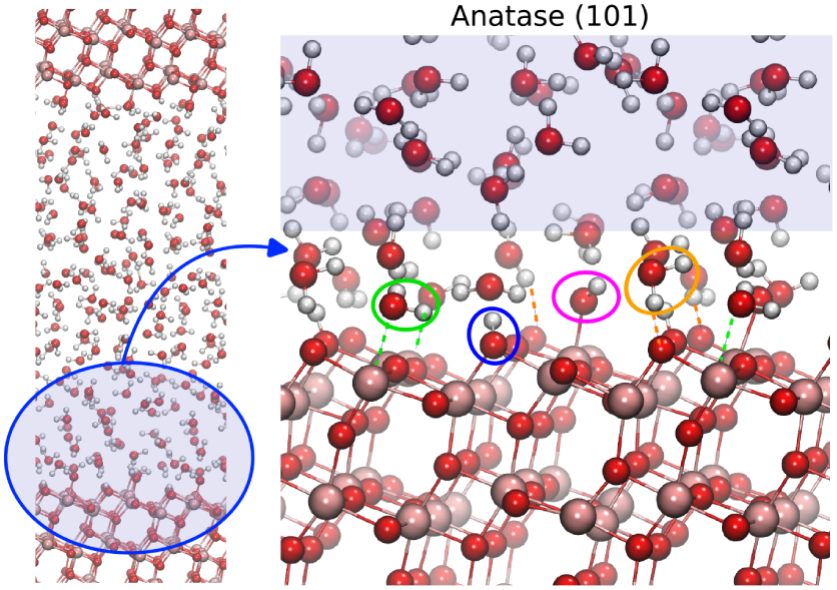
Representative snapshot and magnified view of the simulated anatase (101) TiO_2_ surface. Adsorbed species are indicated: Ti_[5]_–H_2_O (green circles), O_br_–H_2_O (orange circles), Ti_[5]_–OH (magenta circles), and O_br_–H (blue circles) hydroxyl groups. Oxygen atoms are red, hydrogen atoms are white, and titanium atoms are tan. The shadowed blue area represents bulk-like water molecules.

NMR spectra were collected from five samples (S_I_, S_II_, S_III_, S_IV_, and S_V_), each prepared with a different strategy. Sample S_I_ was dried under a high dynamic vacuum (0.001 μbar) at 723.15 K for 40 h and handled inside an argon glovebox. Sample S_II_ was exposed to atmospheric moisture and used ″as is″ without further treatment. Sample S_III_ was treated with a large excess of D_2_O in a sealed autoclave at 500 K for 48 h in order to exchange most of the surface protons with deuterons. Although the excess of D_2_O was evaporated afterward under argon flux, this sample still contained a significant amount of liquid D_2_O/HDO and exhibited a paste-like appearance when packed into the NMR rotor. Solid-state fast MAS NMR (60 kHz) was used to collect the chemical shift signatures from these three samples. Sample S_IV_ was prepared by packing the HR-NMR rotor with titania powder and filling the excess volume of the rotor with liquid D_2_O. Finally sample S_V_ was obtain by further dehydration of sample Sample S_III_. (see Methods)

### Sample S_I_

We start by considering the proton characteristics of the vacuum-dried nanoparticles in the S_I_ sample. The ^1^H MAS NMR spectrum for S_I_ is shown in Figure 3. Two broad resonances are observed at 5.3 and 1.4 ppm, respectively, together with a shoulder at 6.8 ppm and barely discernible signals at 3.0 and −0.3 ppm. The signal at 5.3 ppm indicates that a small amount of water molecules is still present, even after extreme desorption conditions. Spectra collected subsequently, after 5, 10, and 15 h of continuous magic-angle spinning, are also shown and confirm the strongly hydrophilic character of TiO_2_ nanoparticles, the water uptake being indicated by the increase of the peak at 5.3 ppm. The peak maximum also moves slightly toward higher ppm values. This behavior was previously reported by Nosaka et al.^22^ as an indication of the presence of a multilayer structure of water molecules at the surface. It is also in agreement with a more recent study^24^ on increasing water coverage of TiO_2_ interfaces. The presence of a larger shift compared to that of liquid water (4.8 ppm) was interpreted in both studies as the formation of a water network with stronger hydrogen bonding than that in liquid water. In the present study, the intensity of the signal at 1.4 ppm and −0.3 ppm did not change after water uptake. Therefore, we interpret this resonance as originating from the inner layer of water molecules and hydroxyl groups directly in contact with the nanoparticles’ surface.

**Figure 3:**
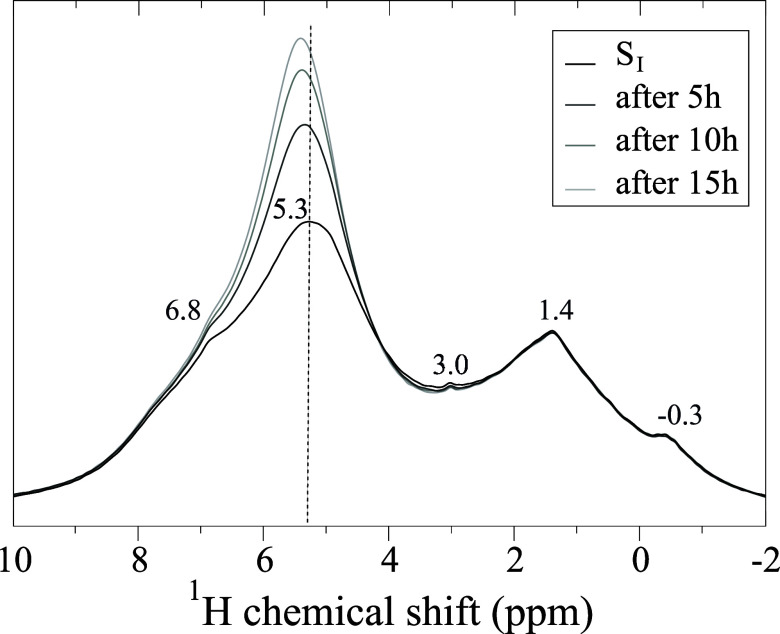
^1^H fast MAS NMR (14.1 T, 60.00 kHz MAS) spectrum of sample S_I_ collected right after the sample was packed into the NMR rotor inside an argon glovebox and after 5, 10, and 15 h of continuous magic-angle spinning.

### Samples S_II_ and S_III_

Next, we consider TiO_2_ nanoparticles exposed to atmospheric moisture and excess heavy water. Figure 4a shows the ^1^H MAS NMR spectra of samples S_II_ and S_III_. The sample S_II_ was only partially hydrated (exposed to atmospheric moisture); therefore, the observed shift in the signal at 5.7 ppm is similar to that of sample S_I_ upon water uptake. On the other hand, the 4.6 ppm peak for sample S_III_ is very close to that expected for liquid HDO (4.8 ppm). The slight difference is attributed to the temperature dependence of the proton chemical shift in liquid HDO, as a consequence of the elevated temperature in the 1.3 mm rotor at 60 kHz MAS (∼320 K).^38^ However, this signal at 4.6 ppm confirms that sample S_III_ contains a substantial excess of liquid-like D_2_O/HDO.

**Figure 4:**
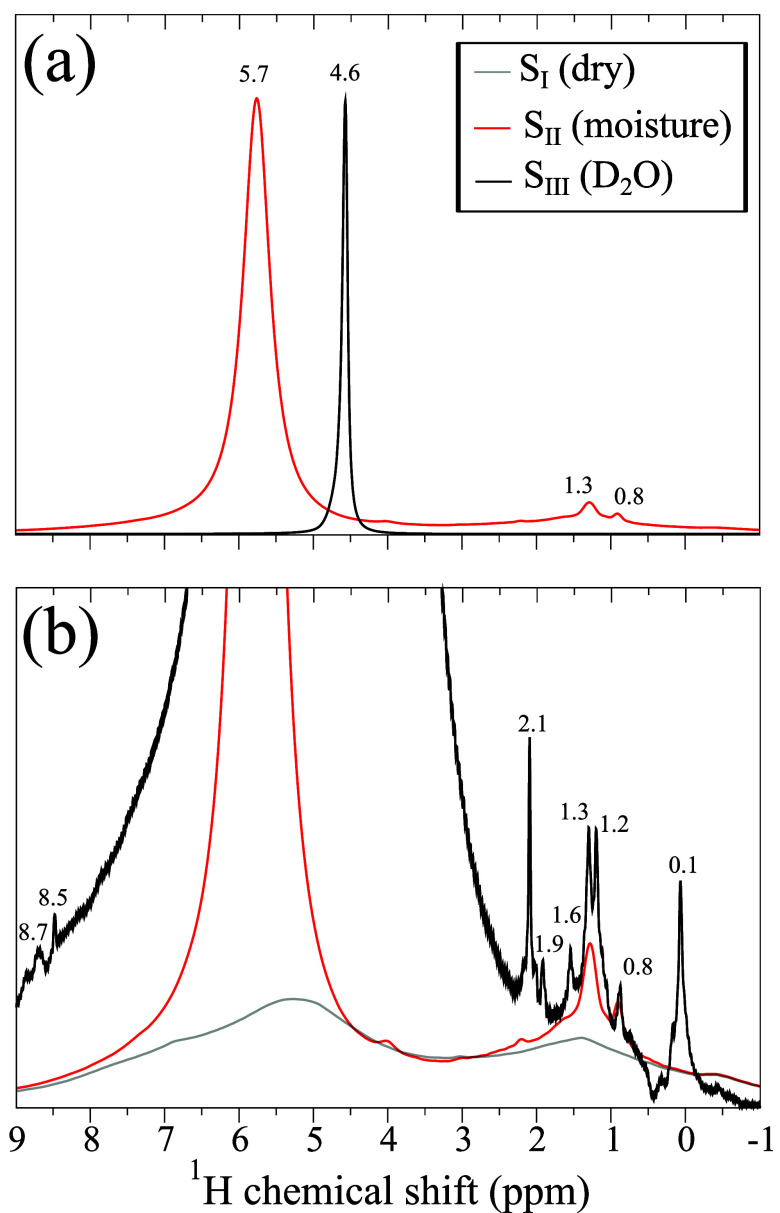
^1^H fast MAS NMR (14.1 T, 60.00 kHz MAS) spectra of samples S_I_, S_II_, and S_III_. Note that in panel (a) the vertical scales are adjusted to match the signal intensity of liquid-like water signal, whereas in panel (b) the spectrum of sample S_III_ is multiplied by a factor of 500.

Figure 4b shows the ^1^H fast MAS NMR spectra of samples S_I_, S_II_, and S_III_ with the 0–2 ppm spectral region emphasized (notably, the spectrum of sample S_III_ is multiplied by a factor of 500). For sample S_II_, the spectrum in the 0–2 ppm range is dominated by a resonance at 1.3 ppm (as previously observed also for TiO_2_ and CeO_2_ nanoparticles),^24,39^ with smaller components around 0.8 ppm also discernible. In contrast, eight sharp peaks are seen for sample S_III_ at 0.1, 0.8, 1.2, 1.3, 1.6, 1.9, 2.1, and 8.5 ppm. We attribute such improved resolution of the ^1^H NMR spectra to the effect of sample deuteration, which eliminates ^1^H homonuclear dipolar couplings because each ^1^H spin will now be surrounded by ^2^H nuclei. The ^2^H MAS NMR spectrum of the same sample of S_III_ is shown in Figure 5. In addition to the resonance of liquid-like D_2_O/HDO at 4.7 ppm, only a weak signal at 2 ppm is seen, indicating that very limited proton-to-deuteron exchange took place at the surface.

**Figure 5:**
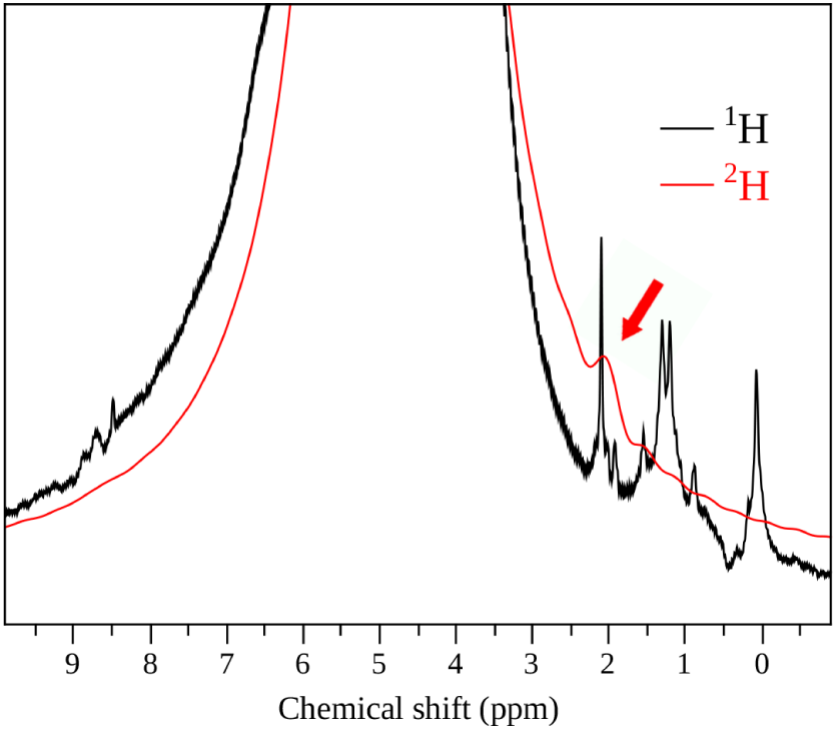
^1^H and ^2^H MAS NMR (14.1 T, 60.00 kHz MAS) spectra collected under identical conditions from the same sample S_III_ prepared with an excess of D_2_O (99.9 atom % D) in a sealed autoclave at 500 K for 48 h.

The intensity of the proton signals in the 0–2 ppm region for sample S_III_ is approximately 2 orders of magnitude weaker compared to S_I_ and S_II_. This is not only a consequence of the scarcity of protons after deuteration but also a result of a much smaller amount of TiO_2_ nanoparticles in the NMR rotor, since a significant sample volume is occupied by the excess of D_2_O/HDO (in contrast to the ″dry″ powders S_I_ and S_II_). Since the efficiency of deuteration of surface species and the precise amount of TiO_2_ nanoparticles in the rotor are not known (paste-like sample appearance, excess of liquid D_2_O/HDO), it is not possible to directly relate the intensities of the NMR ^1^H signal with the surface area of the nanoparticle. We note that we attempted to improve the resolution of proton MAS NMR spectra of TiO_2_ by simply washing and drying the powder with heavy water. However, we could only obtain the same results as shown for the spectrum exposed to moisture, in agreement with similar previous attempts on metal oxide nanoparticles,^25,39^ probably due to ineffective isotope exchange at the surface of the particle and the absence of excess D_2_O in the NMR rotor. Here, the oxide samples are impregnated samples that contain an excess of D_2_O, directly studied in the rotor, which presumably results in more complete deuteration of the water molecules adsorbed at the surface.

### Sample S_IV_

To corroborate our observations, we explored another NMR approach applicable to studies of solid–liquid interfaces, namely, high-resolution nuclear magnetic resonance (HR-NMR). In theory, HR-NMR combines the benefits of high-resolution liquid-state NMR (field homogeneity and stability due to the deuteron lock channel) with the advantages of solid-state MAS NMR (ability to average out dipolar couplings, chemical shift anisotropy, and effects of magnetic susceptibility). In practice, to obtain HR-NMR spectra, the molecules/ligands of interest must undergo significant averaging of anisotropic NMR interactions by molecular motion so that the relatively slow MAS rates of the HR-MAS setup are sufficient to average out the remaining dipolar broadening and magnetic susceptibility effects. Therefore, to facilitate the visibility of surface species at the solid–liquid interface of TiO_2_, a larger amount of D_2_O was used in the S_IV_ sample prepared for HR-NMR experiments. The excess of D_2_O was aimed at allowing for increased mobility of surface species and providing a deuteron lock signal for the HR-NMR probe head.

Comparison of the fast-MAS (60 kHz) NMR proton spectrum of sample S_III_ with the HR-NMR (10 kHz) NMR proton spectrum of sample S_IV_ is reported in Figure 6. All proton resonances observed by fast-MAS NMR for sample S_III_ in the 0–2.1 ppm range were successfully detected by HR-MAS NMR in sample S_IV_. However, the latter revealed slightly lower resolution and somewhat different relative signal intensities, presumably due to a combination of insufficient MAS rate and restricted molecular motion on the surface of TiO_2_ nanoparticles, even with excess liquid D_2_O present in sample S_IV_. Minor differences in chemical shifts between fast-MAS and HR-MAS spectra probably result from temperature effects. Remarkably, detection of resolved proton signals in the 0–2.1 ppm range by HR-MAS under slow MAS for sample S_IV_ (prepared from a different batch of TiO_2_ nanoparticles than sample S_III_) excludes the possibility that these signals could arise from impurities or immobile interstitial hydrogen defects.

**Figure 6:**
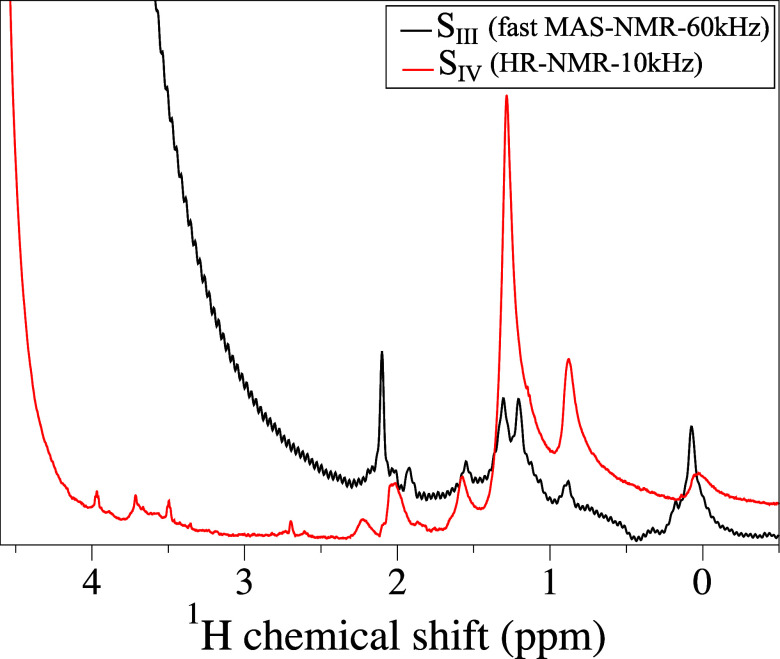
^1^H fast MAS (14.1 T, 60.00 kHz MAS, black trace) and HR-MAS (11.7 T, 10.00 kHz MAS, red trace) NMR spectra of samples S_III_ and S_IV_, respectively.

### Sample S_V_

After establishing the origin of the observed proton resonances, we moved the spotlight onto probing the local connectivity of interfacial water molecules. We mapped proximities among surface species by recoupling proton–proton dipolar couplings in a 2D ^1^H–^1^H double quantum (DQ) → single quantum (SQ) correlation NMR spectrum recorded at 60 kHz MAS, as shown in Figure 7. This experiment was performed on sample S_V_, which corresponded to sample S_III_ subjected to longer evaporation of excess D_2_O until a relatively dry powder was obtained. This was necessary to accommodate more TiO_2_ nanoparticles in the rotor and, in turn, gain enough signal to enable the acquisition of 2D NMR spectrum.

**Figure 7:**
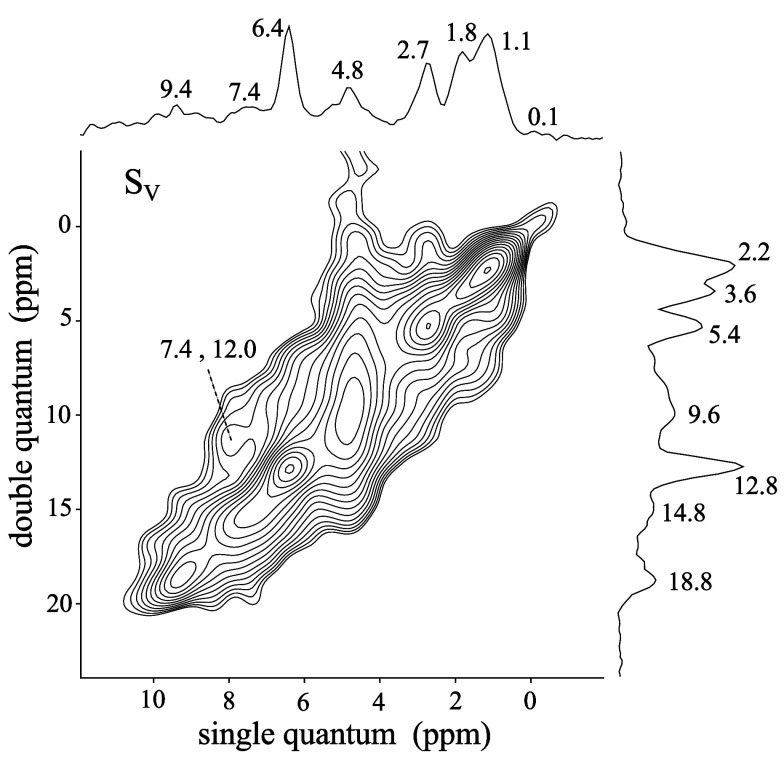
Solid-state ^1^H double quantum (DQ) → single-quantum (SQ) correlation NMR spectrum of sample S_V_, acquired at 60 kHz MAS with a DQ excitation time of 133.3 μs.

In the ^1^H–^1^H DQ → SQ NMR experiment, DQ coherences of dipole-coupled spin pairs appear at the sum of their respective SQ chemical shifts. On-diagonal resonances observed in the spectrum of Figure 7 at 0.1, 1.1, 1.8, 2.7, 4.8, 6.4, and 9.4 ppm (in the SQ dimension) correlate with the respective DQ coherences observed at ∼0.2, 2.2, 3.6, 5.4, 9.6, 12.8, and 18.8 ppm. This autocorrelation reveals mutual dipolar contacts, and therefore close spatial proximities (<5 Å), among protons of the same type. Off-diagonal signals reveal close spatial proximities among chemically distinct types of protons. In the spectrum of Figure 7, the signal observed in the SQ dimension at 7.4 ppm correlates with the off-diagonal signal observed at 12 ppm in the DQ dimension, and at the same time, its on-diagonal autocorrelation is weak. Therefore, this signal can be assigned to hydroxyl groups in contact with ″liquid-like″ water (SQ signal at 4.8 ppm), since 7.4 + 4.8 = 12.2 ppm, and autocorrelation from hydroxyls is expected to be weak due to relatively far distances between neighboring OH groups at the surface. The signal of ″liquid-like″ water at 4.8 ppm (SQ) extends significantly in DQ dimension over the range of 0 to 16 ppm. This indicates off-diagonal features, and therefore contacts, with essentially all observed proton species, except for the resonance observed at 9.4 ppm in the SQ dimension. Observation of dipolar contacts between ″water-like″ liquid and adsorbed species in the ^1^H DQ → SQ NMR spectrum indicates substantially limited molecular mobility of water molecules close to the surface, which preserves the dipolar coupling network. This is in agreement with previous observations of reduced water dynamics under confinement.^37,40^ Signal intensity of the resonance at 4.8 ppm in the ^1^H DQ → SQ spectrum is significantly reduced in comparison to the 1D MAS NMR spectrum of the same sample shown in Figure S2 in the Supporting Information, which shows that water molecules further from the surface exhibit enough rotational/translational dynamics to average out dipolar couplings and hinder detection of DQ coherences. We remark here that proton signals in the 0–2 ppm region were undetected in the presence of liquid water (even using 2D NMR spectroscopy) in previous NMR studies of hydrated oxides.^41,42^

### Theoretical Calculations

Previous studies have generally attributed ^1^H NMR signals in the 0–2.1 ppm region to surface protons that have weak or absent hydrogen bonds.^30,39,41,42^ However, it is not clear whether these resonances originate from hydroxyl groups or from intact water molecules. To shed light on this question, we performed DFT-MD calculations of the ^1^H chemical shifts for the aqueous interface of anatase (101). ^1^H chemical shifts are known to be sensitive to the structure of the water hydrogen-bond network^43^ as well as to geometrical constraints and confinement effects at the surface.^44^ Therefore, four possible systems for the water–TiO_2_ interface were considered: i) a TiO_2_ surface with intact adsorbed water molecules; ii) a TiO_2_ surface with one-third of the adsorbed water molecules in the first layer dissociated into terminal Ti_[5]_–OH and bridging O_br_–H hydroxyl groups (see Figure 2); iii) a TiO_2_ surface with 66% of the Ti_[5]_ adsorption sites occupied by Ti_[5]_–OH groups; and iv) a TiO_2_ surface with 66% of the O_br_ adsorption sites occupied by O_br_–H groups. We note that these protonated surfaces represent the two sides of the same slab so that the entire slab is charge balanced.

For each of these systems, the ^1^H NMR chemical shifts were computed for selected frames of the corresponding DFT-MD trajectory using the GIPAW code^45–48^ and collected into time-averaged histograms (see Methods). In Figure 8, the calculated chemical shifts are reported for a neutral anatase (101) surface with dissociated water (the results for the neutral case with intact water molecules are reported in Figure S4 in Supporting Information). Regardless of the presence of dissociated or intact water molecules, the chemical shifts lie in the range of 3–14 ppm, suggesting that these surface models do not conform to the experimental conditions. The same applies to a surface where only Ti_[5]_–OH groups are present (see Figure S5 in Supporting Information). The agreement between the calculated and experimental spectra was, instead, quite good for the anatase (101) surface where an excess of O_br_–H groups is present. Theoretical analysis in the following is thus focused on this case.

**Figure 8:**
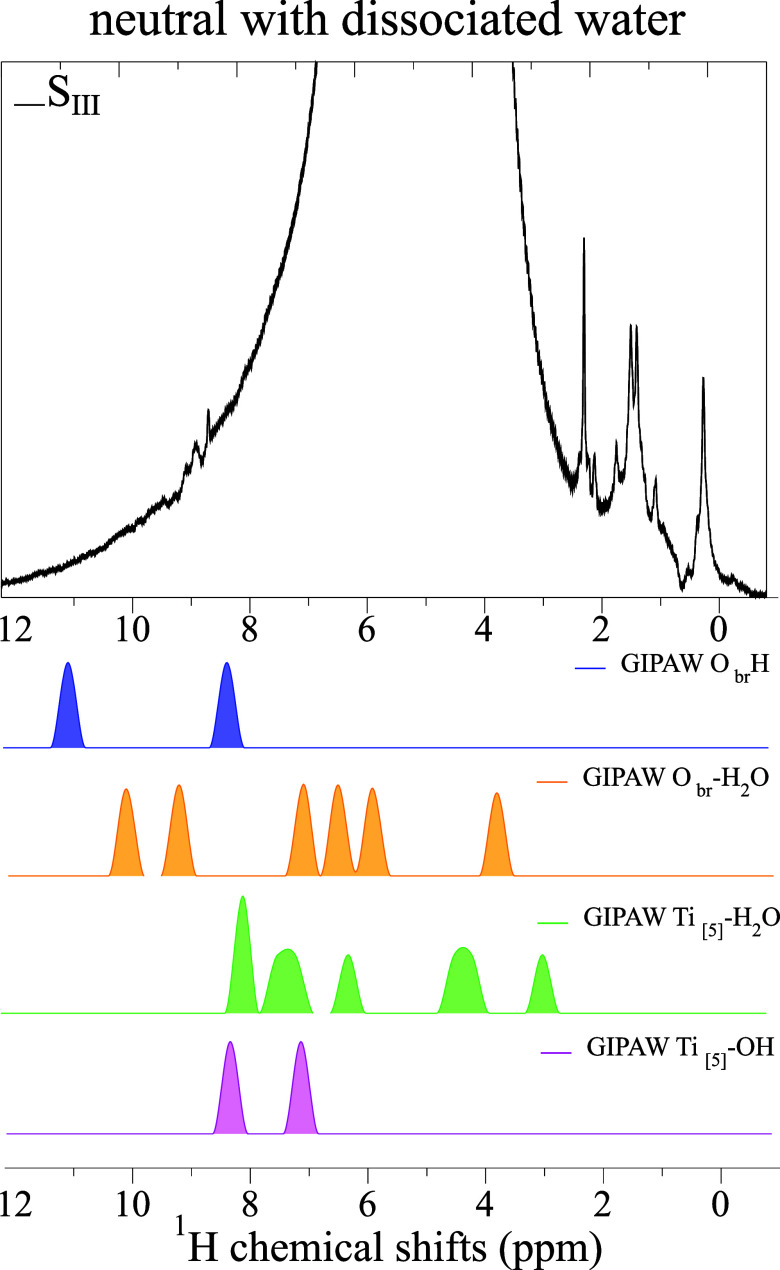
^1^H MAS NMR spectrum of sample S_III_ shown together with the MD-DFT/GIPAW chemical shift histograms for Ti_[5]_–OH, O_br_–H, Ti_[5]_–H_2_O and O_br_–H_2_O on the anatase (101) surface under full hydration (as in Figure 2). Two dissociated water molecules are present on the surface, represented by the two peaks of hydroxyl groups in the 7–12 ppm range. No signature of NMR peaks in the 0–2 ppm range is visible in the GIPAW calculation of this surface, nor when only Ti_[5]_–OH groups are present (see Figure S5 in Supporting Information).

The DFT-MD/GIPAW chemical shift histograms for Ti_[5]_–H_2_O, O_br_–H, and the water molecules in the second hydration layer (O_br_–H_2_O) are compared to the experimental ^1^H MAS NMR spectrum of sample S_III_ in Figure 9. The computed histogram for Ti_[5]_–H_2_O shows two signal components that match the experimentally observed signals at 0.1 and 0.8–1.2 ppm, while the remaining components in the 3–6 ppm range are predicted to be hidden under the signal of liquid water. Inspection of the DFT-MD trajectory further reveals that NMR signals in the 0.1–1.2 ppm range originate from protons of the Ti_[5]_–H_2_O moieties that lack H-bond interactions with neighboring oxygen atoms of H_2_O molecules in the second hydration layer (O_br_–H_2_O). This is illustrated in Figure 10 where the hydrogen bond (H•••O) length distributions are shown for the distinct proton species present on the protonated TiO_2_ surface. It appears that some Ti_[5]_–H_2_O moieties experience much longer hydrogen-bond distances than those in bulk liquid water, and this effect correlates with NMR signals in the 0–2 ppm region. This is in agreement with the experimentally observed chemical shifts of water in weak hydrogen bonding environments, which exhibit resonance in the 0–1.5 ppm region.^49–51^ An illustration of an isolated water molecule surrounded by O_br_–H is displayed in Figure 11a.

**Figure 9:**
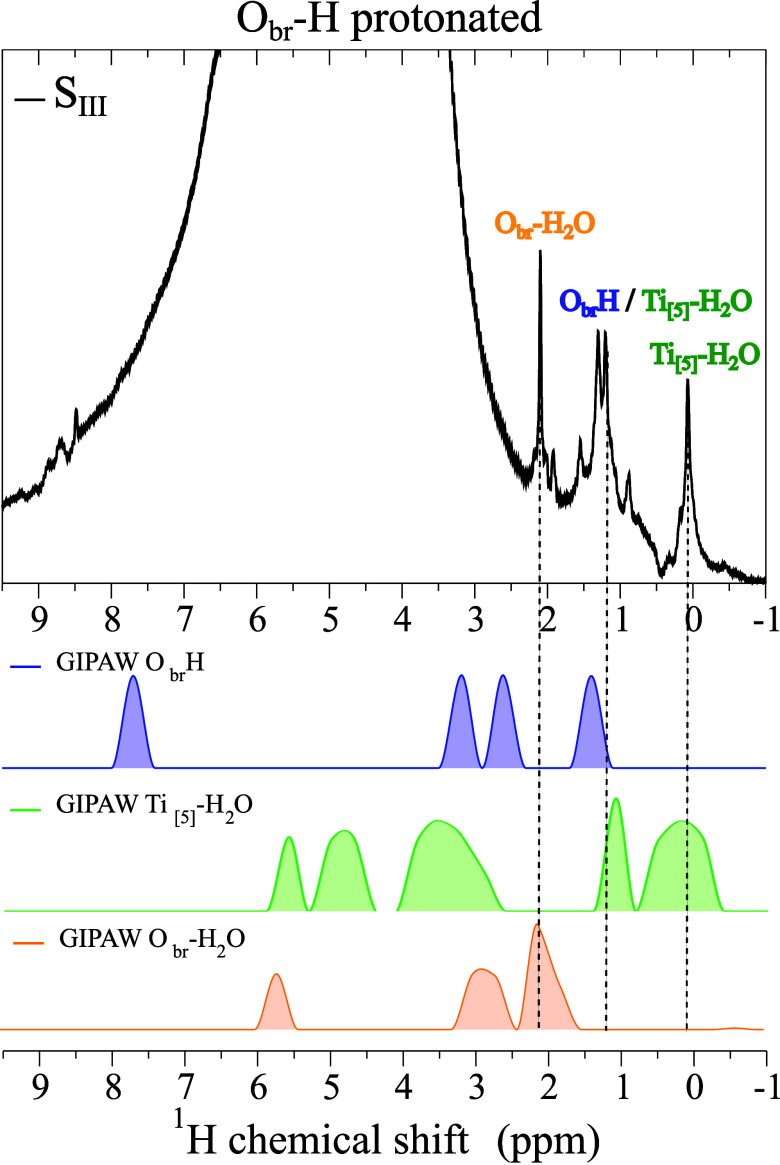
^1^H MAS NMR spectrum of sample S_III_ shown together with the MD-DFT/GIPAW chemical shift histograms for O_br_–H, Ti_[5]_–H_2_O, and O_br_–H_2_O on the anatase (101) surface under full hydration with an excess of O_br_–H groups.

**Figure 10:**
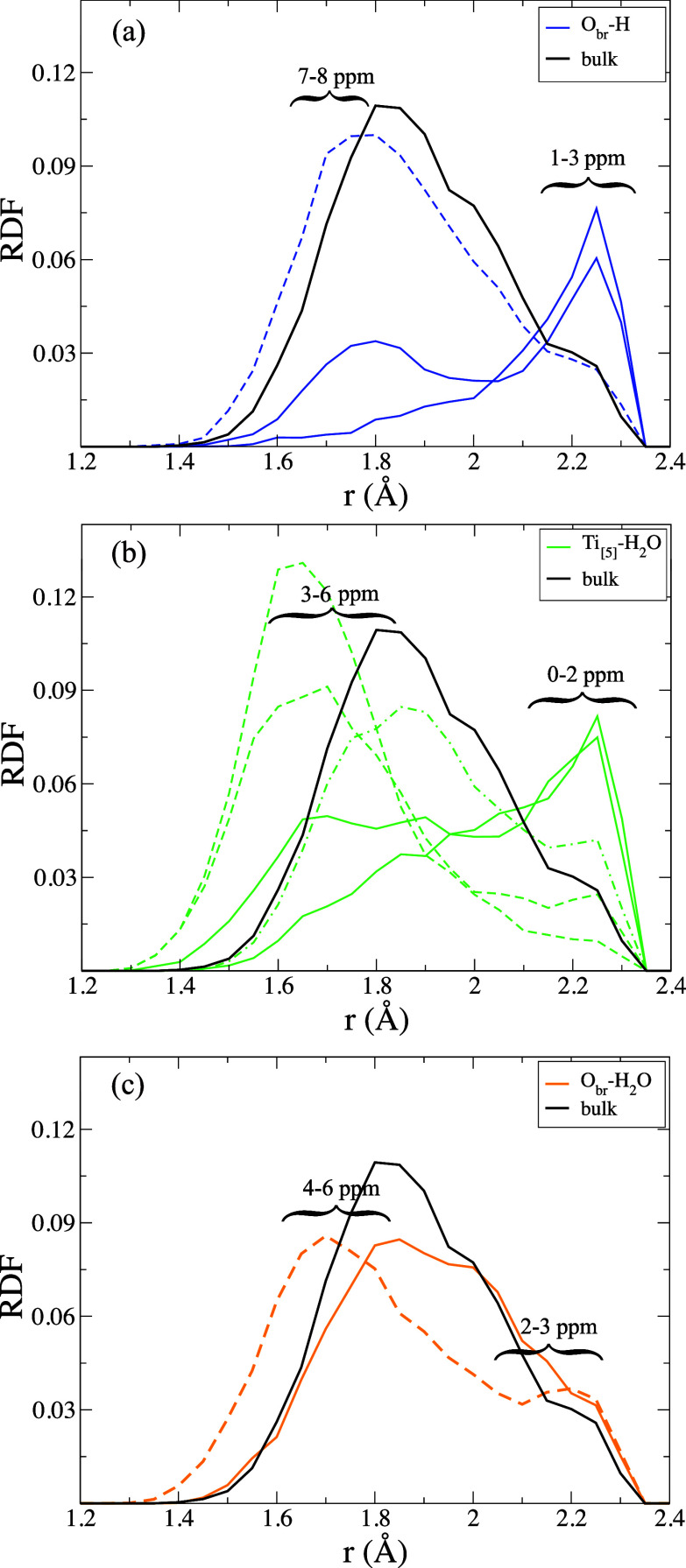
Hydrogen bond (H•••O) length distributions for the interfacial water and hydroxyl groups of the O_br_–H protonated anatase (101) surface as compared with the distribution of bulk water (full black line). The hydrogen bonds are considered between hydrogen atoms in the O_br_–H (a), Ti_[5]_–H_2_O (b) and O_br_–H_2_O (c) species and the surrounding oxygen atoms. Full lines highlight proton species that contribute mostly to the 0–2 ppm region of the NMR spectrum, while dashed lines represent the species that contribute to the 3–8 ppm region. Note that the environment characterized by a longer (H•••O) hydrogen bond network corresponds to lower chemical shift values, indicating weaker (H•••O) interaction. Conversely, a hydrogen bond network shorter than that of bulk water exhibits higher chemical shift values, indicating stronger water–water interactions.

**Figure 11:**
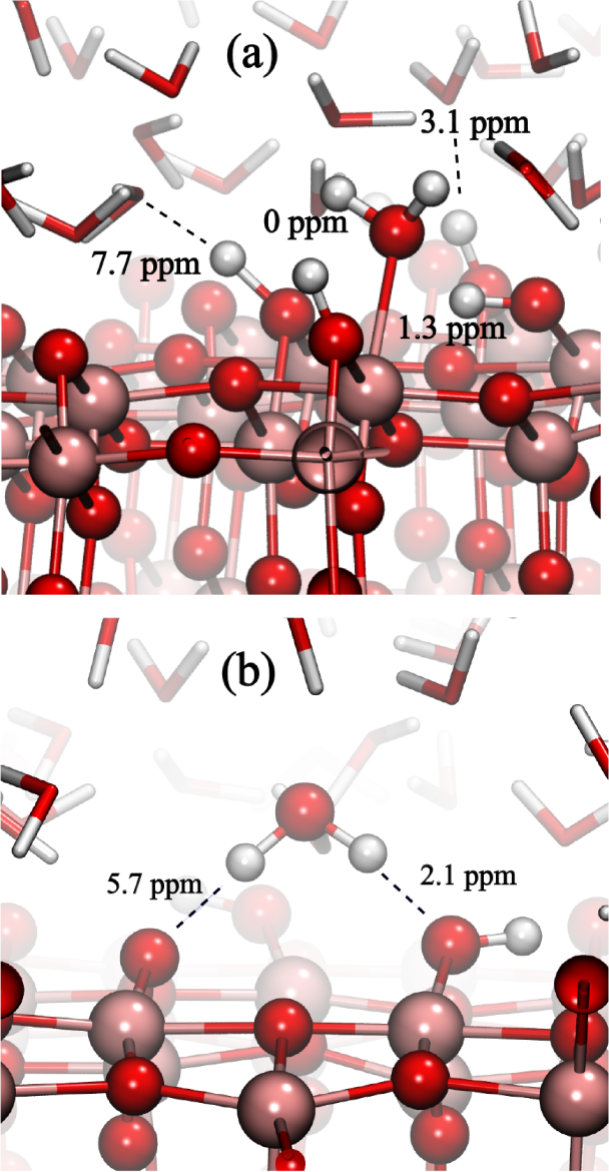
(a) Representative configuration of an isolated Ti_[5]_–H_2_O surrounded by O_br_–H, which gives rise to the signal at 0 ppm. (b) Representative configuration of O_br_–H_2_O when adsorbed on O_br_–H site, which gives rise to the signal at 2.1 ppm.

Although counterintuitive, the presence of dangling water OH groups on a fully hydrated surface can be understood as a result of breaking the hydrogen-bond network induced by the presence of the abundant surface O_br_–H groups. Among the four distinct signal components in the calculated chemical shift histogram of the O_br_–H hydroxyl groups, three can be attributed to hydroxyls that form hydrogen bonds with surrounding water molecules, while the NMR signal at 1.3 ppm originates from more isolated species, whose presence is also evidenced by the distributions of H-bond distances in Figure 10a.

The ^1^H MAS NMR spectrum further shows a peak at 2.1 ppm, also indicating a proton with weak H-bond interactions (Figure 9). Based on the O_br_–H_2_O signal component in the DFT-MD/GIPAW histogram (and the wide distribution of the hydrogen bond lengths for this surface group, Figure 10c), we assign this peak to one of the two protons of O_br_–H_2_O, notably the one that interacts with an O_br_–H, while the second proton interacts strongly with an O_br_ site, as illustrated in Figure 11b. We also note that detection of the distinct NMR signal from O_br_–H_2_O water molecules in the second hydration layer indicates that these do not undergo diffusive exchange but rather show reduced mobility,^37^ possibly as a consequence of their relatively high adsorption energy, comparable to that in the first hydration layer.

To gain more quantitative insight, we performed accurate calculations at the level of domain-based local pair natural orbital coupled cluster theory with single-, double-, and perturbative triple excitations DLPNO-CCSD­(T)^52,53^ for selected representative water motifs extracted from the molecular dynamics trajectory of anatase (101) surface simulation (see Figure S6). Interaction energies of H_2_O molecules with Ti_[5]_ and O_br_ adsorption sites were evaluated with the local energy decomposition analysis (LED).^54^ Our results indicated that the interaction mechanisms involved mainly electrostatics and London dispersion. Although the former is expected to dominate, the extent of the latter is quite surprising. This was especially the case for the O_br_–H_2_O site, which could be regarded as a typical H-bonded system, normally dominated by electrostatics. Specifically, London dispersion accounted for ∼45% and ∼60% of the total interaction energy for the Ti_[5]_–H_2_O and O_br_–H_2_O sites, respectively. The large interaction energies of −69.5 kJ/mol for Ti_[5]_–H_2_O and −61.3 kJ/mol for O_br_–H_2_O (see Figure S6 and Table S1 for details) suggest that these moieties do not participate in exchange/dynamics with the liquid water^37^ and give distinct NMR signals, consistent with the NMR results.

## Discussion

The assignment of proton chemical shifts on dry TiO_2_ interfaces was originally proposed by Crocker et al. in their seminal work.^25^ Based on electron affinity environment arguments, these authors interpreted the resonances in the 1.3 ppm region as generated by Ti_[5]_–OH hydroxyl groups, while the O_br_–H groups were considered to be visible beyond 6 ppm values. The follow-up literature on chemical shift detection of TiO_2_–water interfaces is based on this interpretation, even for hydrated surfaces.^22–24,26,28–30^ In the present work, the assignment of experimental NMR peaks is based on calculated GIPAW chemical shifts of large statistical samples of interfacial water in an aqueous environment. The results support a quite different interpretation in comparison to that of Crocker et al. The only plausible scenario for signals in the 0–2 ppm range is the presence of excess O_br_–H protons, where the signals around 0.1, 1.3, and 2.1 ppm are the effect of O_br_–H groups weakening the H-bonding of the adsorbed water molecules in the Ti_[5]_–H_2_O species. This is also observed in hydrated alumina interfaces,^42^ where weakly interacting hydroxyl groups promote resonances around 2 and 0 ppm. Ti_[5]_–OH hydroxyl group resonance should instead be strongly visible beyond 7 ppm values (for dry samples), as the signals at 7.4 and 9.4 ppm were detected in the 2D DQ–SQ correlation in Figure 7. This assignment is almost opposite to the interpretation present in the literature for TiO_2_–water interfaces and highlights the importance of considering the specific interfacial water structure in aqueous conditions in order to decipher the relative chemical shift environment.

The appearance of ^1^H MAS NMR spectrum of sample S_III_, as well as its interpretation, is also supported by recent studies on LaTiO_2_N photocatalyst.^55^ The fast MAS NMR proton spectrum of LaTiO_2_N revealed surprisingly similar signals at 0.1, 0.8, 1.2, and 2.1 ppm. Interestingly, these signals vanished when La^3+^ was substituted with the paramagnetic Ce^3+^ (CeTiO_2_N). These NMR resonances were broadened (nearly) beyond detection as a result of paramagnetic interactions with unpaired 4*f* electrons of the Ce^3+^ ions, which implies that they correspond to the OH/H_2_O network present directly at the material surface. Since LaTiO_2_N is not as hygroscopic as TiO_2_, all these signals of surface OH/H_2_O species can be observed directly, without special sample preparation. Finally, the chemical shifts of isolated hydroxyl species at around 1.3 ppm as predicted in our MD-DFT/GIPAW calculations for protonated anatase (101) surfaces are in agreement with previous fast MAS NMR surface observations for BaTiO_3_,^56–59^ NdTiO_2_N,^60^ and CaTaO_2_N.^61^ Noteworthy, we also note the existence of a weak signal at around 8.5–8.7 ppm in the spectra of both TiO_2_ (sample S_III_) and LaTiO_2_N and respectively at 7.4 and 9.4 ppm in the ^1^H DQ–SQ spectrum (Figure 7). According to our MD-DFT/GIPAW calculations, proton signals around 8.5 ppm can be assigned to protonated species of dissociated water molecules, both O_br_–H and Ti_[5]_–OH (Figure 8), which interact and autocorrelate in the ^1^H DQ–SQ spectrum. The peak at 7.4 ppm, instead, is attributed to isolated Ti_[5]_–OH of dissociated water, which mostly interacts with adsorbed water molecules.

The fact that the agreement between the experimental NMR spectra and the calculated chemical shift histograms could only be established for the O_br_–H protonated surface model may be attributed to different factors: (i) a local pH below the point of zero charge, in the range of 5.5–5.9, for anatase^62–64^ ; (ii) the presence of hydroxide anions in solution (or in the interfacial water layers); and (iii) the presence of surface oxygen vacancies^18,65^ or reduced Ti sites^66^ which promote the formation of surface O_br_–H groups. For such conditions, our theoretical analysis reveals a weak hydrogen-bond network at the surface, consistent with a hydrophobic character of the O_br_–H protonated anatase–water interface. This aligns with previous studies showing that TiO_2_ is hydrophobic under ambient conditions and becomes hydrophilic only after UV irradiation.^4^ Recent work has attributed this behavior to the selective adsorption of atmospheric formic and acetic acids, which can form a surface monolayer.^67,68^ However, that work focused on rutile and was carried out under ultrahigh vacuum conditions, which is different from the aqueous environment investigated here. In our S_III_ sample, weak signals consistent with the presence of formic and acetic acids were observed at 8.5 and 1.9 ppm, respectively, in agreement with previous ^1^H MAS NMR studies on hydrated biopolymers.^69^ However, their signal intensities are minor compared to those of surface hydroxyl and water species. Furthermore, computational studies suggest that under wet conditions, these acids are unlikely to adsorb directly onto the TiO_2_ surface.^70–72^ Therefore, although their direct contribution to surface hydrophobicity should be minimal, they can influence local pH, indirectly perturbing the hydrogen bond network and enhancing the hydrophobic character through protonation effects.

## Conclusions

Our combined experimental and theoretical study reveals the atomistic complexity of interfacial water on hydrated TiO_2_ nanoparticles. We demonstrate that high-resolution ^1^H MAS NMR spectroscopy, supported by first-principles chemical shift calculations, enables the identification of distinct surface species, including terminal and bridging hydroxyls, as well as hydrogen-bonded and isolated water molecules. In particular, the best agreement between the experiment and simulation is achieved for an O_br_–H protonated anatase (101) surface, which suggests that under ambient conditions, the interface adopts a hydrophobic character and lacks significant populations of dissociated water.

Importantly, this work introduces a powerful methodology for probing atomic-scale features at solid–liquid interfaces under realistic ambient aqueous conditions. The combination of proton-resolved solid-state NMR and first-principles simulations not only yields unprecedented insight into local surface chemistry but also lays the foundation for systematic investigations of a wide range of complex and challenging interfacial aqueous environments, extending beyond TiO_2_ to other catalytic and functional materials in energy, environmental, and biological contexts.

## Methods

### Sample Preparation

Commercial TiO_2_ nanopowder (Sigma-Aldrich 718467; ≥99.5%) was used to prepare all of the samples. Sample S_I_ was placed in Micromeritics ASAP tubes and outgassed ex situ at 723.15 K with a 10 K/min ramp rate under a high dynamic vacuum of 0.001 μbar for 40 h using the degas ports of the Micromeritics ASAP2020 apparatus. Thereafter, the sample was backfilled with 1 atm of dry argon using the dosing manifold of the ASAP2020 device with 2 h of equilibration at room temperature to avoid any leaks during sample transfer. The tube, sealed with an O-ring cap, was transferred to an argon glovebox where the sample was packed into the NMR rotor (Figure S3). Sample S_II_ was exposed to atmospheric moisture and used ″as is″. Sample S_III_ was prepared by immersing 1 g of TiO_2_ nanopowder and 14 mL of D_2_O (Sigma-Aldrich 151882; 99.9 atom % D) in a sealed autoclave that was kept at a temperature of 500 K for 48 h. After evaporation of the excess D_2_O under a flux of argon, the wet powder was packed into the NMR rotor under ambient conditions. Sample S_IV_ was prepared by using a different batch of the same TiO_2_ nanopowder (Sigma-Aldrich 718467; ≥99.5%). The powder was directly packed into the HR-NMR rotor and immersed in a large excess of liquid D_2_O. Sample S_V_ was obtained by the same procedure used for sample S_III_, but the evaporation of the excess liquid D_2_O was prolonged until obtaining a relatively dry powder.

### TiO_2_ Nanopowder Characterization

The morphology of TiO_2_ nanopowder was evaluated by using high-resolution transmission electron microscopy and electron diffraction on a JEOL 2100F field emission transmission electron microscope operated at 200 kV. X-ray powder diffraction pattern was collected with a Panalytical X’Pert PRO MPD diffractometer using monochromatic X-ray radiation (λ = 1.5406 Å). The structural models of anatase and rutile were refined against the data using TOPAS 6 software.^73^

### Solid-State NMR

Solid-state ^1^H fast MAS NMR spectra were acquired at the facilities of Stockholm University by using a magnetic field strength of 14.1 T (Larmor frequency of 600.1 MHz for ^1^H) with a Bruker Avance III spectrometer. Acquisitions were performed with a 1.3-mm MAS probe head at an MAS rate of 60.00 kHz. The rotor-synchronized, double-adiabatic spin–echo sequence with a 90-deg excitation pulse of 1.1 μs, followed by two 50.0 μs tanh/tan short high-power adiabatic pulses^74,75^ with a 5 MHz frequency sweep was used to provide a flat baseline and suppress probe/rotor background signals in the spectra. All pulses operated at a nutation frequency of 220 kHz. 256 signal transients were accumulated for samples S_I_ and S_II_, whereas 16384 signal transients were collected for the deuterated sample S_III_. A relaxation delay of 5 s was used in all 1D experiments. The 2D ^1^H–^1^H DQ → SQ correlation NMR spectrum of sample S_V_ was acquired at 60 kHz MAS and with 133.3 μs of DQ excitation time (8 rotor periods) using Back-to-Back (BaBa) homonuclear recoupling pulse sequence at a nutation frequency of 220 kHz.^76^ 128 *t*_1_ increments were recorded, each with 2048 signal transients using a relaxation delay of 0.5 s. ^1^H chemical shifts were referenced with respect to tetramethylsilane (TMS). Dry air with a dew point of < −90 °C (relative humidity <0.00%) was used as a drive and bearing gas for MAS. The magic angle was set by recording ^79^Br MAS NMR spectra of solid KBr at 60.00 kHz MAS and measuring the width of the first sideband to be <250 Hz.

### High-Resolution NMR

High-resolution ^1^H MAS NMR spectra were acquired at Princeton University using a magnetic field strength of 11.7 T (Larmor frequency of 500.0 MHz for ^1^H) with a Bruker Avance III spectrometer. Acquisitions were performed with a 4.0-mm rotor at a MAS rate of 10.00 kHz. The pulse sequence was “zg”, a single-pulse experiment. The 90-deg pulse width was 3 μs, and the relaxation delay was 5 s. The number of scans was 4096 at a temperature of 298.2 K.

### Computational Methods

MD-DFT simulations were performed using the CP2K software package.^77^ The TiO_2_ slabs contained 4 × 3 × 3 unit cells^15^ of anatase (101), and the simulation box was set to be 10.55 × 11.40 × 43.00 Å. The vacuum region of about 33 Å was filled with H_2_O molecules at the water density at 1 atm and 310 K. The simulations were run for 20 ps in an NVT ensemble, where the temperature was controlled by the Bussi thermostat.^78^ The BLYP DFT functional^79,80^ with the D3 dispersion correction^81^ was used together with GTH pseudopotentials^82,83^ and a polarized double-ζ Gaussian basis set (DZVP)^84^ for the valence electrons. An energy cutoff of 370 Ry was employed for plane waves. The hydroxylated neutral anatase (101) surface was prepared to have 40% of the Ti_[5]_ and O_br_ sites occupied by split water molecules, generating the Ti_[5]_–OH and O_br_–H hydroxyl groups. We observed recombination during the simulation of one water molecule, leading to an occupancy of 33% of the Ti_[5]_ sites. This surface was used in conjunction with the previously published^15^ trajectory of the neutral anatase (101) surface. The protonated anatase (101) surfaces were obtained by placing four OH and four H atoms on the respective sides of the anatase slab to maintain the charge neutrality of the simulation box. From each equilibrium trajectory, 40 frames were selected for NMR calculations, which were performed with the GIPAW^45,46^ module of the Quantum Espresso software package.^47,48^ A very tight convergence threshold of 1 × 10^–10^ *E*_h_ was used with the PBE functional^85,86^ a single (Γ) point in the Brillouin zone, and cutoff energies of 80 and 800 Ry for plane waves and electron density, respectively. For each proton, we collected the time-averaged ^1^H NMR shielding on a histogram with a bin value of 0.7 ppm. Since the GIPAW method is known to have a resolution for proton shifts of about 0.3 ppm,^87,88^ the results are well within the error bar of the computation. The calculated nuclear magnetic shielding constants were converted to NMR chemical shifts using reference shieldings obtained from a simulation of liquid water (128 H_2_O) performed with the same computational setup as for simulations of TiO_2_ surfaces, assuming an experimental reference chemical shift for liquid water of 4.6 ppm. In order to assign the calculated chemical shifts to specific hydroxyl surface species, we identified different OH proton surface species, namely, Ti–OH_2_, O_br_–H, Ti–OH, and O_br_–H_2_O. This was achieved by defining each species through the O–Ti and O–H coordination numbers, having distance cutoffs of 2.3 and 1.2 Å, respectively. Specifically, Ti–OH_2_ is defined by oxygens one-coordinated with Ti and two-coordinated with H, O_br_–H by oxygens two-coordinated with Ti and one-coordinated with H, Ti–OH by oxygens one-coordinated with Ti and one-coordinated with H, and O_br_–H_2_O by oxygens zero-coordinated with Ti (free water) and H–O_br_ coordination within 1.2–2.1 Å. For each proton of the identified species, we collected the histograms of the associated chemical shifts as well as their radial distribution function from the neighboring oxygen atoms.

DLPNO-CCSD­(T)-LED calculations were performed with the ORCA code^89,90^ (see Supporting Information for more details). More information is available from the corresponding authors upon request.

## Supplementary Material



## Notes

X-ray powder diffraction; Additional solid-state NMR data on sample S_V_; Additional MD-DFT/GIPAW NMR shift histograms of the (101) anatase TiO_2_ with either intact molecular adsorbed water molecules or a Ti_[5]_–OH protonated surface; Details on DLPNO–CCSD­(T)-LED and GGA calculations (PDF)
