# Supplementary material for: Atomic-Scale Mapping of Interfacial Water on Oxide Surfaces via Proton-Resolved NMR and Ab Initio Simulations
Source: J Am Chem Soc. 2026 Mar 16;148(11):11583–94. doi: 10.1021/jacs.5c18863 (PMC13022891; doi:10.1021/jacs.5c18863)
Supplement: Supplementary file 1 [file ja5c18863_si_001.pdf]

Supporting Information for: Atomic-Scale Mapping of Interfacial Water on Oxide Surfaces via Proton-Resolved NMR and Ab-Initio Simulations

Lorenzo Agosta,[*,†] Ken Conover,[‡] Przemyslaw Rzepka,[¶] Alisa Gordeeva,[§] Adam Slabon,[‖] Istvan Pelczer,[‡] Annabella Selloni,[‡] Kersti Hermansson,[†] and Aleksander Jaworski[*,§]

†*Department of Chemistry, Ångström Laboratory, Uppsala University, 751 21 Uppsala, Sweden*

‡*Department of Chemistry, Princeton University, Princeton, New Jersey 08544, USA*

¶*J. Heyrovsky Institute of Physical Chemistry, Czech Academy of Sciences, 18223 Prague, Czech Republic*

§*Department of Chemistry, Stockholm University, 106 91 Stockholm, Sweden*

‖*Chair of Inorganic Chemistry, Bergische Universität Wuppertal, 42119 Wuppertal, Germany*

E-mail: lorenzo.agosta@kemi.uu.se; aleksander.jaworski@su.se

X-ray powder diffraction pattern

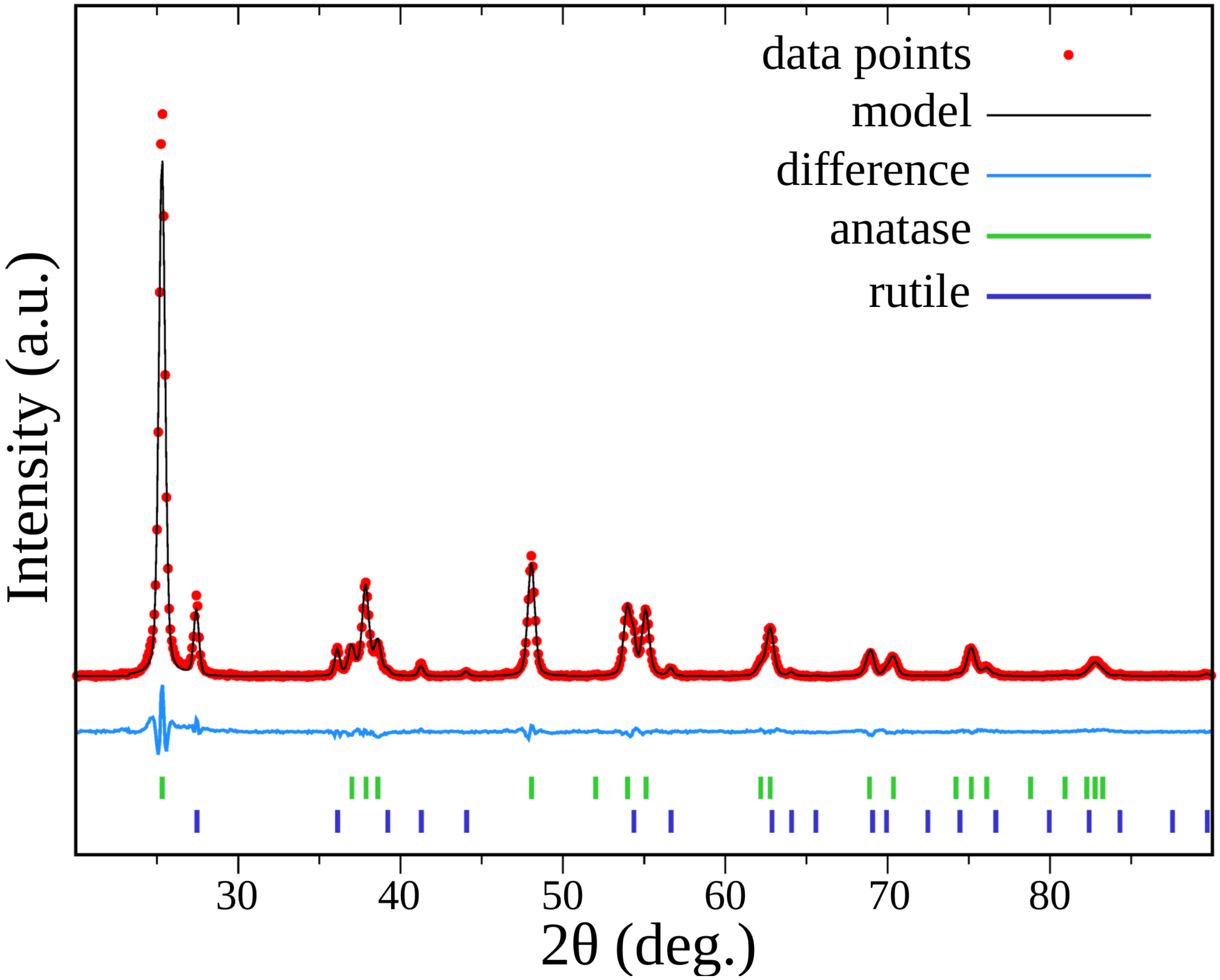

Figure S1: X-ray powder diffraction pattern; Rietveld analysis provided anatase/rutile percentage ratio of 88/12 (±1).

Additional solid-state NMR data on sample S_V

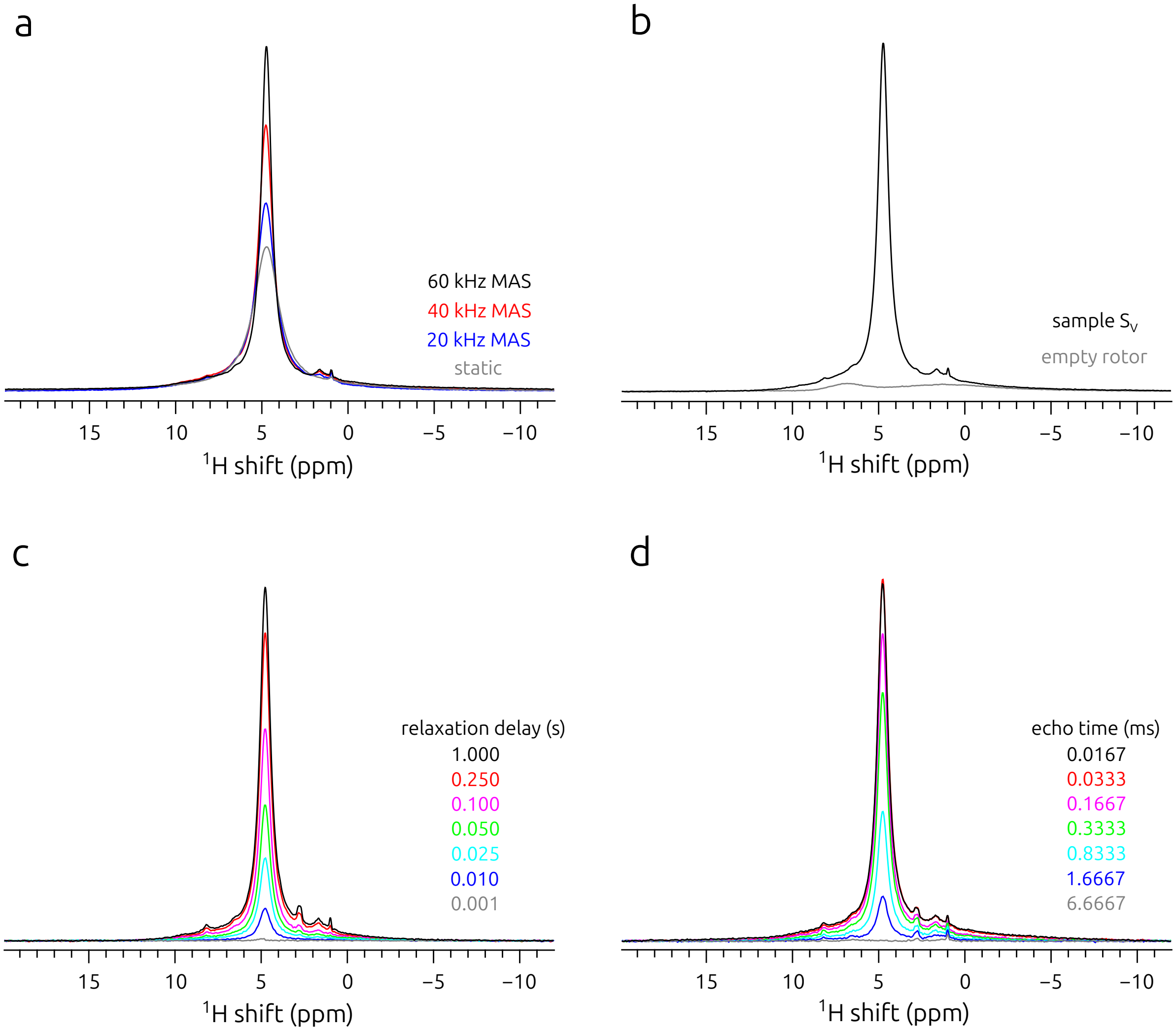

Figure S2: Comparison of ^{1}H MAS NMR spectra of sample S_V collected at different MAS rates using double-adiabatic spin-echo sequence with two 50 μs SHAP pulses, which corresponds to 3, 2, and 1 rotor period at MAS rate of 60, 40, and 20 kHz, respectively (panel a). ^{1}H MAS NMR spectrum of sample S_V at 60 kHz MAS compared with that of empty rotor collected under identical conditions and same number of scans (panel b). ^{1}H longitudinal relaxation (T_1) tests for sample S_V recorded with Hahn-echo sequence with total echo time of 10 rotor periods and with saturation pulse-train (panel c). ^{1}H transverse relaxation (T_2) tests for sample S_V recorded with Hahn-echo sequence and 1 s relaxation delay (panel c)

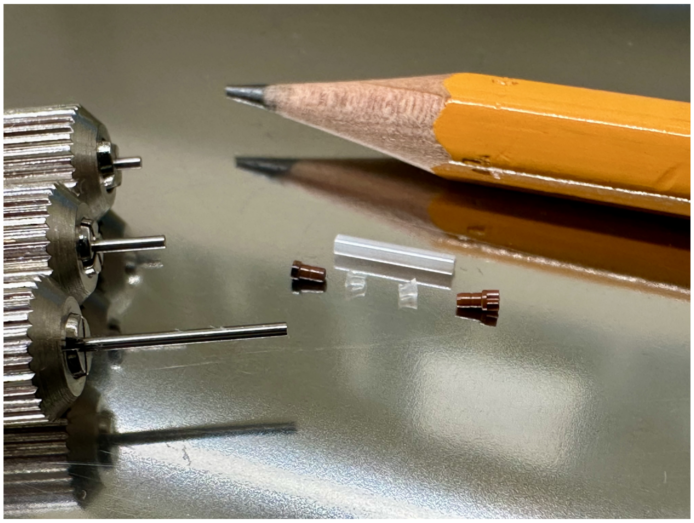

Figure S3: 1.3mm MAS rotor used for all fast MAS experiments and shown together with the drive- and the bottom-cap, as well as with the sealing inserts. When the rotor spins, centrifugal forces expand slightly sealing inserts in radial direction providing sealing effect.

Additional MD-DFT/GIPAW NMR shifts histograms

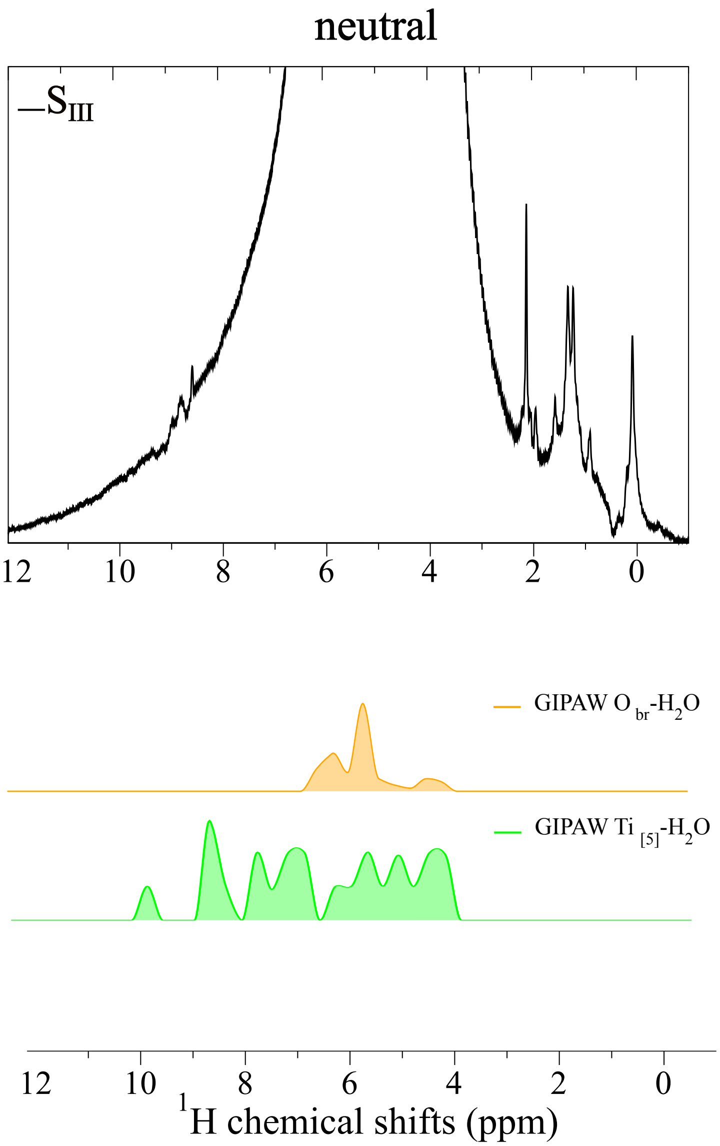

Figure S4: MD-DFT/GIPAW NMR chemical shifts histograms shown together with the spectrum of sample S_{III} for a TiO_2 anatase (101) surface with only intact adsorbed water molecules.

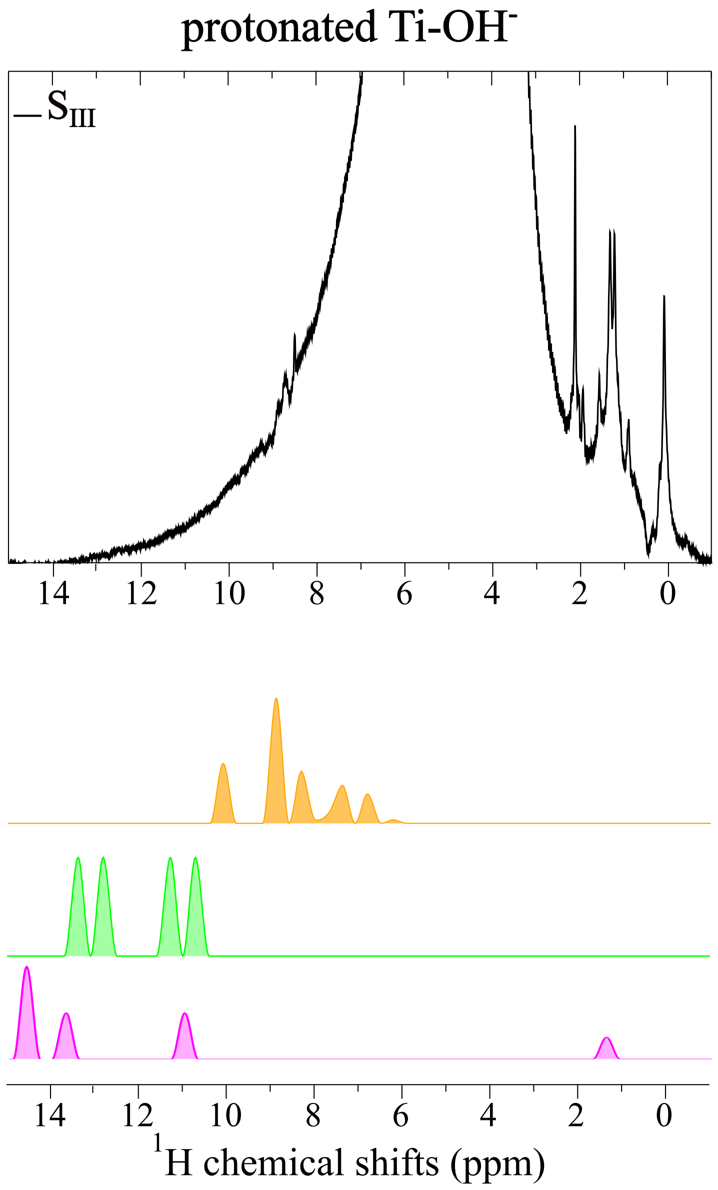

Figure S5: MD-DFT/GIPAW NMR chemical shifts histograms shown together with the spectrum of sample S_{III} for a TiO_2 anatase (101) surface with $Ti_{[5]}{-}OH$ groups.

DLPNO-CCSD(T)-LED calculations

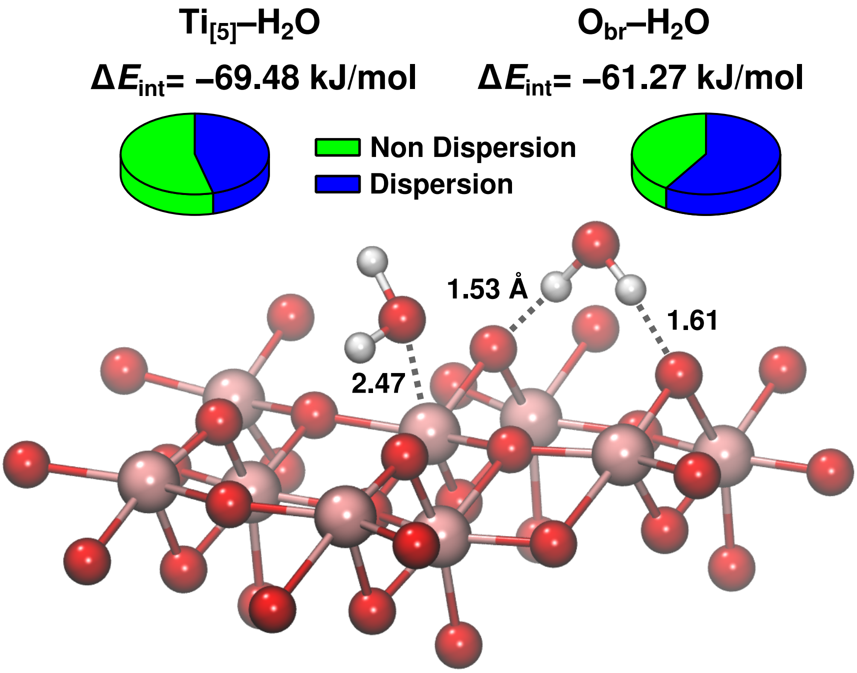

Figure S6: The model of anatase (101) surface with adsorbed $Ti_{[5]}-H_2O$ and $O_{br}-H_2O$ species used for DLPNO-CCSD(T)-LED local energy decomposition analysis. Interaction energies at each adsorption site are indicated, as well as the partitioning of dispersive and non-dispersive contrubitions to the binding energy.

Interaction energy of H_2O molecule in contact with TiO_2 surface at the given adsorption site $Ti_{[5]}-H_2O$ (1st layer) or $O_{br}-H_2O$ (2nd layer) was evaluated with local energy decomposition analysis (LED),[1,2] within the scheme proposed recently by Jaworski and Hedin.[3] This allowed to decompose the interaction energy into meaningful physical terms, which altogether result in the total interaction energy $\Delta E_{\rm int}$; see Table S1.
The large electronic preparation contribution $\Delta E^{\rm HF}_{\rm el-prep}$ corresponded to the energy needed to bring the electronic structures of the isolated fragments into the one optimal for the interaction. For both $Ti_{[5]}-H_2O$ and $O_{br}-H_2O$ the $\Delta E^{\rm HF}_{\rm el-prep}$ was counteracted by substantial electrostatic ($E_{\rm elstat}$) and smaller exchange ($E_{\rm exch}$) attractive contributions. The repulsive non-dispersive corrections due to electron correlation ($\Delta E^{\rm C}_{\rm non-disp}$) were counteracted by attractive contributions resulting from London dispersion ($E^{\rm C}_{\rm disp}$) and corrections to the interaction energy for the triple excitations ($\Delta E^{\rm C-(T)}_{\rm int}$). The summed Hartree-Fock ($\Delta E^{\rm HF}_{\rm el-prep} + E_{\rm elstat} + E_{\rm exch}$; $\Delta E^{\rm HF}_{\rm int}$) and correlation ($\Delta E^{\rm C}_{\rm non-disp} + E^{\rm C}_{\rm disp} + \Delta E^{\rm C-(T)}_{\rm int}$; $\Delta E^{\rm C}_{\rm int}$) components of the interaction energy were negative (attractive) for both $Ti_{[5]}-H_2O$ and $O_{br}-H_2O$. The non-dispersive contributions due to electron correlation ($\Delta E^{\rm C}_{\rm non-disp}$) were considerably repulsive for both $Ti_{[5]}-H_2O$ and $O_{br}-H_2O$, and provided corrections to the permanent electrostatic interaction calculated at the Hartree-Fock level, which tends to over-

Table S1. Results of the DLPNO-CCSD(T)-LED local energy decomposition analysis (in kJ/mol) for $Ti_{[5]}-H_2O$ and $O_{br}-H_2O$ species at the anatase (101) surface.

Contribution	$Ti_{[5]}-H_2O$	$O_{br}-H_2O$
$\Delta E^{HF}_{el-prep}$	+317.06	+673.56
E_{elstat}	−309.23	−603.49
E_{exch}	−50.20	−102.47
$\Delta E^{C}_{non-disp}$	+6.57	+9.60
E^{C}_{disp}	−31.62	−35.94
$\Delta E^{C-(T)}_{int}$	−2.05	−2.53
ΔE^{HF}_{int}	−42.38	−32.40
ΔE^{C}_{int}	−27.10	−28.86
ΔE_{int}	−69.48	−61.27
$E^{C}_{disp}/\Delta E_{int}$	0.46	0.59

estimate the dipole moments.[2] Consequently, the interaction mechanisms could be assumed to involve mainly electrostatics and London dispersion. Whereas the former was expected to dominate because of permanent dipole moment of the H_2O molecule, the extent of the latter was surprising. That was especially the case for the $O_{br}-H_2O$ site, which could be regarded as a typical H-bonded system, normally dominated by electrostatics. However, London dispersion accounted to 46% and 59% of the total interaction energy for the $Ti_{[5]}-H_2O$ and $O_{br}-H_2O$ sites, respectively, whereas it is only 27% for the water dimer in comparison (as an example of a model H-bonded system).[1]
DLPNO-CCSD(T)-LED calculations were performed with the ORCA code[4,5] using very tight convergence tolerance of $1x10^{-9}$ E_h. `TightPNO` interfragment, and `NormalPNO` intrafragent truncation settings were used together with a full local MP2 guess. RIJCOSX approximation with def2/J Coulomb-fitting basis set[6] and tight grid (`GridX7`) were employed. The revised Los Alamos National Laboratory triple-ζ basis set augmented with f-polarization (LANL2TZ(f))[7] was employed for Ti, and correlation-consistent triple-ζ cc-pVTZ basis set[8] for O and H, respectively, together with cc-pVTZ/C auxiliary basis set.[9] Calculations were performed for the model derived from the representative molecular dynamics trajectory snapshot frame of anatase (101) surface simulation. Oxygen atoms at the bottom side of the cluster were terminated with protons in order to provide charge balance.

To inspect the reliability of the GGA-DFT-D3 level of theory employed in MD-DFT simulations (see main text for simulation parameters), we calculated the interaction energies between the full TiO_2 slab and the H_2O molecules in Fig. S6 for the same trajectory frame from which the model for DLPNO-CCSD(T)-LED calculations was derived.

Interaction energies ($\Delta E_{int}^{GGA-DFT-D3}$) were calculated according to:

$$\Delta E_{int}^{GGA-DFT-D3} = E_{\mathrm{TiO_2}(slab)+\mathrm{H_2O}} - E_{\mathrm{TiO_2}(slab)} - E_{\mathrm{H_2O}}$$

where $E_{\mathrm{TiO_2}(slab)+\mathrm{H_2O}}$ was the total energy of the system while $E_{\mathrm{TiO_2}(slab)}$ and $E_{\mathrm{H_2O}}$ were those for the isolated $TiO_{2(slab)}$ slab and the respective H_2O molecules at molecular geometries corresponding to the $E_{\mathrm{TiO_2}(slab)+\mathrm{H_2O}}$ system. The obtained $E_{int}^{GGA-DFT-D3}$ interaction energies were −75.01 and −53.03 kJ/mol for the adsorption on the Ti_5 and the O_{br} site, respectively. These compared well with accurate estimates from the DLPNO-CCSD(T) level of theory, which in turn indicated that the GGA-DFT-D3 approximation used in our MD-DFT simulations was capable to grasp the essential physics of the studied system.

References

(1) Schneider, W. B.; Bistoni, G.; Sparta, M.; Saitow, M.; Riplinger, C.; Auer, A. A.; Neese, F. Decomposition of Intermolecular Interaction Energies within the Local Pair Natural Orbital Coupled Cluster Framework. *J. Chem. Theory Comput.* **2016**, *12*, 4778–4792.

(2) Altun, A.; Neese, F.; Bistoni, G. Effect of Electron Correlation on Intermolecular Interactions: A Pair Natural Orbitals Coupled Cluster Based Local Energy Decomposition Study. *J. Chem. Theory Comput.* **2019**, *15*, 215–228.

(3) Jaworski, A.; Hedin, N. Local energy decomposition analysis and molecular properties of encapsulated methane in fullerene (CH_4@C_{60}). *Phys. Chem. Chem. Phys.* **2021**, *23*, 21554–21567.

(4) Neese, F. The ORCA Program System. *Wiley Interdiscip. Rev. Comput. Mol. Sci.* **2012**, *2*, 73–78.

(5) Neese, F.; Wennmohs, F.; Becker, U.; Riplinger, C. The ORCA Quantum Chemistry Program Package. *J. Chem. Phys.* **2020**, *152*, 224108.

(6) Weigend, F. Accurate Coulomb-Fitting Basis Sets for H to Rn. *Phys. Chem. Chem. Phys.* **2006**, *8*, 1057–1065.

(7) Roy, L. E.; Hay, P. J.; Martin, R. L. Revised Basis Sets for the LANL Effective Core Potentials. *J. Chem. Theory Comput.* **2008**, *4*, 1029–1031.

(8) Dunning, T. H. Gaussian Basis Sets for Use in Correlated Molecular Calculations. I. The Atoms Boron Through Neon and Hydrogen. *J. Chem. Phys.* **1989**, *90*, 1007–1023.

(9) Weigend, F.; Köhn, A.; Hättig, C. Efficient Use of the Correlation Consistent Basis Sets in Resolution of the Identity MP2 Calculations. *J. Chem. Phys.* **2002**, *116*, 3175–3183.
